# Accumulation of Antibiotic Resistance Genes in Carbapenem-Resistant *Acinetobacter baumannii* Isolates Belonging to Lineage 2, Global Clone 1, from Outbreaks in 2012–2013 at a Tehran Burns Hospital

**DOI:** 10.1128/mSphere.00164-20

**Published:** 2020-04-08

**Authors:** Masoumeh Douraghi, Johanna J. Kenyon, Parisa Aris, Mahla Asadian, Sedighe Ghourchian, Mohammad Hamidian

**Affiliations:** a Division of Microbiology, Department of Pathobiology, School of Public Health, Tehran University of Medical Sciences, Tehran, Iran; b Institute of Health and Biomedical Innovation, Queensland University of Technology, Brisbane, Queensland, Australia; c The ithree institute, University of Technology Sydney, Ultimo, New South Wales, Australia; Escola Paulista de Medicina/Universidade Federal de São Paulo

**Keywords:** AbaR4, *Acinetobacter baumannii*, GC1, Iran, Tehran, Tn*2006*, antibiotic resistance, carbapenem resistance, genomic island, global clone 1, *oxa23*, *oxa72*

## Abstract

The worldwide distribution of carbapenem-resistant Acinetobacter baumannii (CRAB) has become a global concern, particularly in countries where antibiotic prescription is not tightly regulated. However, knowledge of the genomic aspects of CRAB from many parts of the world is still limited. Here, 50 carbapenem-resistant A. baumannii isolates recovered at a single hospital in Tehran, Iran, during several outbreaks in 2012 and 2013 were found to be resistant to multiple antibiotics. They were examined using PCR mapping and multilocus sequence typing (MLST). All Iranian strains belonged to sequence type 328 in the Institut Pasteur MLST scheme (ST328_IP_), a single-locus variant of ST81_IP,_ and all Iranian strains contained two carbapenem resistance genes, *oxa23* and *oxa24.* The *oxa23* gene is in the transposon Tn*2006* in AbaR4, which interrupts the chromosomal *comM* gene. Phylogenetic analysis using whole-genome sequence (WGS) data for 9 isolates showed that they belonged to the same clade, designated the ST81/ST328 clade, within lineage 2 of global clone 1 (GC1). However, there were two groups that included either KL13 or KL18 at the K locus (KL) for capsular polysaccharide synthesis and either a *tet39* or an *aadB* resistance gene, respectively. The genetic context of the resistance genes was determined, and the *oxa24* (OXA-72 variant) and *tet39* (tetracycline resistance) genes were each in a p*dif* module in different plasmids. The *aadB* gene cassette (which encodes gentamicin, kanamycin, and tobramycin resistance) was harbored by pRAY*, and the *aphA6* gene (which encodes amikacin resistance) and *sul2* gene (which encodes sulfamethoxazole resistance) were each harbored by a different plasmid. The sequences obtained here will underpin future studies of GC1 CRAB strains from the Middle East region.

**IMPORTANCE** Carbapenem-resistant Acinetobacter baumannii strains are among the most critical antibiotic-resistant bacteria causing hospital-acquired infections and treatment failures. The global spread of two clones has been responsible for the bulk of the resistance, in particular, carbapenem resistance. However, there is a substantial gap in our knowledge of which clones and which specific lineages within each clone are circulating in many parts of the world, including Africa and the Middle East region. This is the first genomic analysis of carbapenem-resistant A. baumannii strains from Iran. All the isolates, from a single hospital, belonged to lineage 2 of global clone 1 (GC1) but fell into two groups distinguished by genes in the locus for capsule biosynthesis. The analysis suggests a potential origin of multiply antibiotic-resistant lineage 2 in the Middle East region and highlights the ongoing evolution of carbapenem-resistant GC1 A. baumannii strains. It will enhance future studies on the local and global GC1 population structure.

## Abstract (precis)

Carbapenem-resistant Acinetobacter baumannii strains are among the most critical antibiotic-resistant bacteria causing hospital-acquired infections and treatment failures. The global spread of two clones has been responsible for the bulk of the resistance, in particular, carbapenem resistance. However, there is a substantial gap in our knowledge of which clones and which specific lineages within each clone are circulating in many parts of the world, including Africa and the Middle East region. This is the first genomic analysis of carbapenem-resistant A. baumannii strains from Iran. All the isolates, from a single hospital, belonged to lineage 2 of global clone 1 (GC1) but fell into two groups distinguished by genes in the locus for capsule biosynthesis. The analysis suggests a potential origin of multiply antibiotic-resistant lineage 2 in the Middle East region and highlights the ongoing evolution of carbapenem-resistant GC1 A. baumannii strains. It will enhance future studies on the local and global GC1 population structure.

## INTRODUCTION

Acinetobacter baumannii is a Gram-negative opportunistic pathogen that causes a range of nosocomial infections. It has become a major global threat because of its high level of resistance to a wide range of antibiotics, which often complicates treatment and leads to treatment failure (1–4). Most A. baumannii isolates resistant to all or most of the antibiotics currently used for treatment belong to one of two major globally distributed clones, known as global clone 1 (GC1) and global clone 2 (GC2) (5, 6).

Carbapenem antibiotics are active against most β-lactamase-producing organisms, including those with extended-spectrum β-lactamase enzymes (7). They are the antibiotics of choice and are considered frontline treatment for infections caused by multidrug-resistant bacteria (8), but alarmingly, the rate of carbapenem resistance is increasing among A. baumannii isolates, imposing huge financial and health care burdens (1, 9–11). Indeed, carbapenem-resistant A. baumannii (CRAB) has emerged as one of the biggest challenges in the treatment of infections caused by this organism, especially when it involves GC1 or GC2 isolates that are already resistant to a wide range of alternative antibiotics (12, 13). Among several carbapenem resistance genes identified so far, *oxa23* appears to be the most widespread in A. baumannii, regardless of clonal type (12).

In the last decade, there have been a number of studies reporting the emergence and rise of resistance to multiple antibiotics, including high levels of carbapenem resistance in different geographic regions of Iran (14–16). In 2012 and 2014, two separate studies reported high incidences of carbapenem resistance caused by *oxa23* in isolates that belong to GC1 and GC2 (17, 18). Recently, a systematic review examining the rate of CRAB estimated an overall ∼80% rate of resistance to carbapenems across the country, with 73%, 21%, and 6.2% of carbapenem-resistant isolates containing the *oxa23*, *oxa24*, and *oxa58* oxacillin carbapenemase genes, respectively (14). However, to date, none of the studies investigated the genetic context and/or genomic location of the *oxa23* gene, which is the most widely encountered carbapenem resistance gene in the country (14) and globally (19).

In 2015, we reported the resistance profiles of 401 clinical A. baumannii isolates recovered from 5 hospitals in Iran between 2011 and 2013, with 90% of the isolates being found to be extensively drug resistant (XDR) (20). Later, all the isolates were further examined using allele-specific PCRs (21), and 57 (14%) and 86 (22%) isolates were found to belong to GC1 and GC2, respectively (22). It was also shown that the majority of multiply drug-resistant isolates, including all GC1 and GC2 strains, contained an interrupted *comM* gene (22). This location is where AbaR-type (23) and AbGRI1-type (24) resistance islands are often present in members of the GC1 and GC2 clonal complexes, respectively. Interruption of *comM* in the Iranian isolates therefore provided evidence that they might carry a resistance island in the *comM* gene (22). Interestingly, 50 out of 57 GC1 isolates were isolated during several outbreaks in 2012 and 2013 at a single hospital (hospital H5) (22).

Here, we sought to further examine this set of 50 GC1 outbreak isolates to investigate the distribution of sequence types (STs) and class D carbapenemase genes and the structure(s) of the resistance island (RI) occupying the *comM* gene. The whole-genome sequences (WGS) of 9 representative strains were also determined to examine the phylogenetic relationship of representative isolates by comparing them to known GC1 isolates from different countries that belong to the defined GC1 lineages, lineage 1 and lineage 2.

## RESULTS

### PCR screening, resistance island mapping, and resistance profiles.

**(i) Identification of GC1 isolates and antibiotic resistance profiles.** All 50 GC1 isolates (Table 1) were previously examined (22) and identified to be GC1 using the allelic-specific PCR described previously (21). Here, representatives from each ward were also tested using PCR and sequencing and found to carry an allele that encodes an OXA-69 variant of the intrinsic A. baumannii oxa (*oxa-Ab*) gene, consistent with their assignment to GC1, as previously described (5, 6). They were all found to be resistant to multiple antibiotics, including ampicillin, streptomycin, spectinomycin, sulfonamides, trimethoprim, ceftazidime, cefotaxime, ticarcillin-clavulanate (Timentin), ceftriaxone, imipenem, meropenem, doripenem, ciprofloxacin, nalidixic acid, and kanamycin (see Table S1 in the supplemental material). Forty-nine strains were resistant to amikacin (only strain ABS230 exhibited complete susceptibility), and 33 strains were resistant to tetracycline (Table S1). All were defined to be extensively drug resistant (XDR), based on the criteria defined previously (nonsusceptibility to one agent in all but two antibiotic classes or less) (25). The complete antibiotic resistance profiles (28 antibiotics) are included in Table S1.

**TABLE 1:** Properties of GC1 A. baumannii isolates recovered in an Iranian burns hospital (hospital H5) in 2012-2013

Isolate^*a*^	Yr	Site	Ward	Presence of:															
				IS*Aba1*- *ampC*	*aadB* in pRAY*	J1	J3	J5	J6	J4	J2	*comM*- Tn	*tniB-* *tniE*	*tniE*- IS*Aba1*	*sup-* *oxa23*	IS*Aba1*- *oxa23*	*oxa23*- IS*Aba1*	*oxa23*- orf4	*oxa23-* *comM*
**ABS029**	**2012**	**Wound**	**2**	+	−	+	−	−	−	−	+	+	+	+	+	+	+	+	+
ABS035	2012	Wound	2	+	+	+	−	−	−	−	+	+	+	+	+	+	+	+	+
**ABS042**	**2012**	**Wound**	**ICU**^*b*^	+	+	+	−	−	−	−	+	+	+	+	+	+	+	+	+
ABS045	2012	Wound	2	+	−	+	−	−	−	−	+	+	+	+	+	+	+	+	+
ABS046	2012	Wound	2	+	+	+	−	−	−	−	+	+	+	+	+	+	+	+	+
**ABS062**	**2012**	**Wound**	**Pediatric**	+	−	+	−	−	−	−	+	+	+	+	+	+	+	+	+
**ABS063**	**2012**	**Wound**	**Emergency**	+	−	+	−	−	−	−	+	+	+	+	+	+	+	+	+
ABS064	2012	Wound	Pediatric	+	−	+	−	−	−	−	+	+	+	+	+	+	+	+	+
**ABS078**	**2013**	**Wound**	**Emergency**	+	+	+	−	−	−	−	+	+	+	+	+	+	+	+	+
ABS081	2013	Wound	Pediatric	+	+	+	−	−	−	−	+	+	+	+	+	+	+	+	+
ABS083	2013	Wound	ICU	+	+	+	−	−	−	−	+	+	+	+	+	+	+	+	+
ABS084	2013	Wound	Pediatric	+	−	+	−	−	−	−	+	+	+	+	+	+	+	+	+
ABS085	2013	Wound	2	+	+	+	−	−	−	−	+	+	+	+	+	+	+	+	+
ABS086	2013	Wound	Pediatric	+	+	+	−	−	−	−	+	+	+	+	+	+	+	+	+
ABS087	2013	Wound	ICU	+	+	+	−	−	−	−	+	+	+	+	+	+	+	+	+
ABS094	2013	Wound	ICU	+	+	+	−	−	−	−	+	+	+	+	+	+	+	+	+
ABS101	2013	Wound	2	+	−	+	−	−	−	−	+	+	+	+	+	+	+	+	+
**ABS103**	**2013**	**Wound**	**ICU**	+	+	+	−	−	−	−	+	+	+	+	+	+	+	+	+
**ABS104**	**2013**	**Blood**	**2**	+	+	+	−	−	−	−	+	+	+	+	+	+	+	+	+
ABS105	2013	Wound	1	+	+	+	−	−	−	−	+	+	+	+	+	+	+	+	+
ABS115	2013	Wound	2	+	+	+	−	−	−	−	+	+	+	+	+	+	+	+	+
ABS121	2013	Wound	Pediatric	+	−	+	−	−	−	−	+	+	+	+	+	+	+	+	+
**ABS122**	**2013**	**Wound**	**1**	+	−	+	−	−	−	−	+	+	+	+	+	+	+	+	+
ABS124	2013	Wound	Pediatric	+	−	+	−	−	−	−	+	+	+	+	+	+	+	+	+
ABS138	2013	Wound	2	+	+	+	−	−	−	−	+	+	+	+	+	+	+	+	+
ABS155	2013	Wound	2	+	+	+	−	−	−	−	+	+	+	+	+	+	+	+	+
ABS178	2013	Wound	2	+	+	+	−	−	−	−	+	+	+	+	+	+	+	+	+
ABS180	2013	Wound	ICU	+	+	+	−	−	−	−	+	+	+	+	+	+	+	+	+
ABS186	2013	Wound	1	+	+	+	−	−	−	−	+	+	+	+	+	+	+	+	+
**ABS201**	**2013**	**Wound**	**Pediatric**	+	−	+	−	−	−	−	+	+	+	+	+	+	+	+	+
ABS206	2013	Wound	1	+	+	+	−	−	−	−	+	+	+	+	+	+	+	+	+
ABS216	2013	Wound	ICU	+	+	+	−	−	−	−	+	+	+	+	+	+	+	+	+
ABS219	2013	Wound	Pediatric	+	−	+	−	−	−	−	+	+	+	+	+	+	+	+	+
ABS224	2013	Wound	ICU	+	−	+	−	−	−	−	+	+	+	+	+	+	+	+	+
ABS226	2013	nr^*c*^	nr	+	+	+	−	−	−	−	+	+	+	+	+	+	+	+	+
ABS230	2013	Wound	ICU	+	+	+	−	−	−	−	+	+	+	+	+	+	+	+	+
ABS237	2013	Wound	2	+	+	+	−	−	−	−	+	+	+	+	+	+	+	+	+
ABS249	2013	Wound	ICU	+	+	+	−	−	−	−	+	+	+	+	+	+	+	+	+
ABS256	2013	Wound	1	+	−	+	−	−	−	−	+	+	+	+	+	+	+	+	+
ABS258	2013	Wound	ICU	+	+	+	−	−	−	−	+	+	+	+	+	+	+	+	+
ABS260	2013	Wound	2	+	+	+	−	−	−	−	+	+	+	+	+	+	+	+	+
ABS263	2013	Wound	2	+	+	+	−	−	−	−	+	+	+	+	+	+	+	+	+
ABS267	2013	Wound	2	+	−	+	−	−	−	−	+	+	+	+	+	+	+	+	+
ABS274	2013	Wound	ICU	+	−	+	−	−	−	−	+	+	+	+	+	+	+	+	+
ABS278	2013	Wound	2	+	+	+	−	−	−	−	+	+	+	+	+	+	+	+	+
ABS283	2013	Wound	Pediatric	+	+	+	−	−	−	−	+	+	+	+	+	+	+	+	+
ABS285	2013	Wound	2	+	+	+	−	−	−	−	+	+	+	+	+	+	+	+	+
ABS288	2013	Wound	ICU	+	+	+	−	−	−	−	+	+	+	+	+	+	+	+	+
ABS290	2013	Wound	2	+	+	+	−	−	−	−	+	+	+	+	+	+	+	+	+
ABS294	2013	Wound	2	+	+	+	−	−	−	−	+	+	+	+	+	+	+	+	+

a All strains carried the *oxa23* and *oxa24* class D carbapenem resistance genes, and all strains belonged to ST328_IP_ in the Institut Pasteur MLST scheme. Results for isolates recovered in 2012 are shaded gray. The whole-genome sequences of the strains shown in bold were determined in this study.

b ICU, intensive care unit.

c nr, not recorded.

10.1128/mSphere.00164-20.1 TABLE S1 Antibiotic resistance profiles of GC1 strains. Download Table S1, DOCX file, 0.04 MB. Copyright © 2020 Douraghi et al. 2020 Douraghi et al. This content is distributed under the terms of the Creative Commons Attribution 4.0 International license.

**(ii) Antibiotic resistance genes.** In all isolates, a copy of IS*Aba1* was detected upstream of the chromosomal *ampC* gene (Table 1), accounting for their resistance to 3rd-generation cephalosporins, consistent with the role of IS*Aba1* in increasing the expression of the *ampC* gene (26, 27). All 50 strains carried the *oxa23* and *oxa24* carbapenem resistance genes (Table 1), accounting for their resistance to carbapenems, as well as ticarcillin-clavulanate. All isolates but ABS230 were resistant to amikacin, and we recently showed that these isolates carry the *aphA6* gene in Tn*aphA6* (28), consistent with this phenotype. All isolates were also resistant to sulfonamide compounds, and consistent with this profile, a copy of the *sul2* gene was found in them.

Thirty-four strains were resistant to tobramycin, in addition to gentamicin and kanamycin (Table S1), and consistent with this phenotype, the *aadB* gene (which encodes gentamicin, kanamycin, and tobramycin resistance) was detected in these strains (Table 1). The *aadB* cassette was not in a class 1 integron but was instead found by PCR to be in the location that it occupies in the small plasmid pRAY (29). Though 33 strains were found to be tetracycline resistant, here, a copy of the *tetA*(A) and *tetA*(B) tetracycline resistance genes was not detected in any strain. However, the genome sequencing data for 5 tetracycline-resistant strains determined (see below) here revealed a copy of the *tet39* resistance gene. Hence, we screened by PCR the entire set and found a 100% correlation between the presence of *tet39* and the tetracycline resistance phenotype in all 33 tetracycline-resistant strains (PCR data not shown).

**(iii) All outbreak isolates carry the *oxa23* gene in AbaR4, located in *comM*.** We previously showed that the *comM* gene was interrupted in all 50 GC1 isolates recovered in hospital H5 (22). Here, PCR was used to screen these strains for general features of the AbaR0/3-type resistance islands that are often found in the chromosomal *comM* gene in GC1, including the class 1 integron, AbaR junctions with *comM* (junctions J1 and J2) (5, 6), and the AbaR internal junctions (junctions J3 to J6) (5, 6), as well as resistance genes frequently found in AbaRs [*arsB*, *aphA1b*, *catA1*, *tetA*(A), and *bla*_TEM_] (23). Unexpectedly, none of the features associated with AbaR-type RIs, including a class 1 integron with the *sul1* gene and the associated *aacC1*-orfP-orfP-orfQ-*aadA1* gene cassettes, the cadmium/zinc transposon Tn*6018*, and the *ars* operon (encoding arsenic resistance), were detected in any isolates. However, among the AbaR0/3-type junctions (J1 to J6), only J1 and J2 produced the expected amplicons, suggesting that a genomic island with a backbone related to that of AbaR0/3-type islands (such as Tn*6021*, Tn*6022*, or AbaR4, which has a Tn*6022* transposon backbone) might be present in the *comM* gene of these Iranian outbreak isolates.

**FIG 1:**
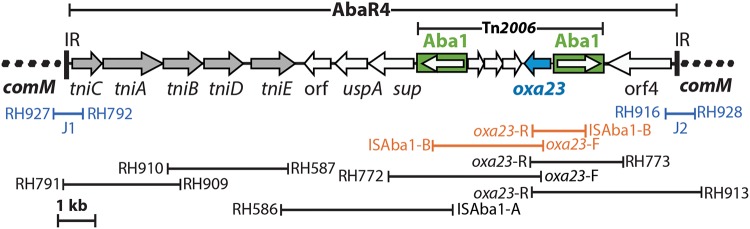
PCR mapping strategy used to identify AbaR4 in the *comM* gene. The interrupted chromosomal region (the *comM* gene) flanking AbaR4 is shown as dashed lines, and inverted repeats (IRs) of AbaR4 are shown as vertical lines. The extents of Tn*2006* and AbaR4 are indicated at the top. IS*Aba1* copies are shown as filled boxes colored green, with the arrows inside them indicating the orientation of the transposition genes. The arrows on the thick central line indicate the extent and orientation of the genes and the open reading frames (ORF). The PCR primers used for mapping are shown as horizontal lines at the bottom, with the primer names being given adjacent to the lines. The figure was drawn to scale using the SnapGene Viewer (v4.1.9) and Adobe Illustrator CS6 (v16.0.3) programs.

The boundaries between *comM* and the genomic island (the J1 and J2 junctions) were amplified and sequenced for strain ABS201, which was randomly chosen as a representative. Sequence analysis revealed that these amplicons were identical to the corresponding junctions of the AbaR4 island previously described in D36, an Australian GC1 lineage 2 strain that carries the *oxa23* gene in Tn*2006* located in AbaR4, which interrupts the chromosomal *comM* gene (30). Using a PCR mapping strategy developed previously (30, 31), we found a complete copy of AbaR4 located in *comM* in all 50 isolates by linking *oxa23* to *comM* (Fig. 1) and detecting the central portion of the Tn*6022*/AbaR4 transposons (Table 1). This finding raised the possibility that all 50 outbreak strains might be related to D36, a strain that contains AbaR4 in *comM* and that was recovered from a member of the military who had returned to Australia (30, 31). The fact that D36 belongs to lineage 2 within GC1 (10) suggested that all Iranian strains may also belong to the same lineage.

**(iv) MLST.** The sequence type (ST) in the Institut Pasteur multilocus sequence typing (MLST) scheme (ST_IP_) (32) was determined using PCR and sequencing for all 50 Iranian isolates. All strains were found to belong to ST328 (*cpn60-1*, *fusA1*, *gltA1*, *pyrG25*, *recA5*, *rplB1*, and *rpoB2*), which is a single-locus variant (SLV) of ST81 (*cpn60-1*, *fusA1*, *gltA1*, *pyrG1*, *recA5*, *rplB1*, and *rpoB2*), which includes isolate D36. ST81 is a double-locus variant of ST1 (*cpn60-1*, *fusA1*, *gltA1*, *pyrG1*, *recA5*, *rplB1*, and *rpoB1*), which represents the majority of strains that belong to GC1 lineage 1. ST328 and ST81 differ by only 2 nucleotides in the *pyrG* gene (a *pyrG1* allele type in ST81 versus a *pyrG25* allele in ST328). ST328 is a rare sequence type, with only one representative in the Institut Pasteur MLST database (http://pubmlst.org/abaumannii/), isolate 67 from Tehran, Iran, suggesting that ST328 might be commonly found in the country.

### Whole-genome analysis and comparison of genomes.

**FIG 2:**
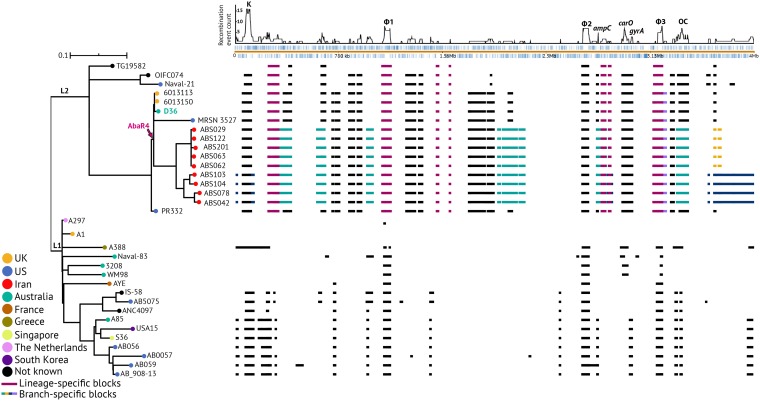
Phylogenetic tree of representatives of Iranian isolates compared to known GC1 strains. L1 and L2 indicate lineages 1 and 2, respectively, and the tree scale bar is shown. The lineage 1 and lineage 2 nodes as well as the nodes including the D36 and Iranian strains have 100% bootstrap support. Recombination blocks within the GC1 genomes across the A1 reference genome (GenBank accession no. CP010781) are indicated using filled boxes, and the plot at the top shows the density of recombination events (number of SND per site) detected against the reference sequence. Lineage 2-specific blocks are colored magenta, and branch-specific blocks within lineage 2 are shown in green, yellow, and blue. Color-coded nodes indicate the country of isolation, and the pink arrow marked AbaR4 indicates the insertion point of this genetic element in lineage 2. Black nodes indicate strains for which information on the country of isolation is not available. The SND scale bar indicates the number of SND per site.

**(i) Placing the Iranian isolates in the global GC1 phylogeny.** To examine if the Iranian outbreak strains fall within lineage 2 of GC1, the whole-genome sequences of 9 representative strains (the strains shown in bold in Table 1) were determined using the Illumina MiSeq technology (Table 2) and used to construct a recombination-free phylogenetic tree in combination with other GC1 isolates previously determined to belong to lineage 1 and lineage 2 (Fig. 2) (10), including the A1 (lineage 1) (33) and D36 (lineage 2) (31) genomes. Two further ST81 strains (strains PR332 and MRSN 3527; Table 3), found by screening the entire NCBI GenBank and whole-genome shotgun databases (as of July 2019), were also included in the phylogenetic analysis. The resulting phylogeny (Fig. 2) demonstrated that all 9 Iranian strains clustered together, branching from the D36 subclade, indicating that they all belong to lineage 2 of GC1. The two ST81 strains (PR332 and MRSN 3527) were also found to belong to lineage 2. Analysis of the single nucleotide differences (SNDs) across the genomes indicated that, on average, the Iranian strains differed from D36 by 50 SNDs, indicating a close relationship. The complete SNDs between all strains analyzed here can be found in Table S2.

**TABLE 2:** Genome sequence data statistics for Iranian GC1 isolates

Isolate	Assembly length (bp)	No. of contigs	No. of read pairs	Read depth (mean fold)^*a*^	*N*_50_^*b*^ (kbp)	GenBank accession no.
ABS029	4,102,995	106	1,370,402	197	101	WIOH00000000
ABS042	4,172,082	106	1,370,826	149	140	WIOG00000000
ABS062	4,104,184	116	1,371,953	107	102	WIOF00000000
ABS063	4,102,168	109	1,367,413	195	102	WIOE00000000
ABS078	4,183,937	97	1,369,455	125	145	WIOD00000000
ABS103	4,244,771	107	1,368,430	170	150	WIOC00000000
ABS104	4,136,827	102	1,370,892	144	150	WIOB00000000
ABS122	4,102,555	108	1,367,643	113	102	WIOA00000000
ABS201	4,198,099	125	1,365,667	115	90	VJZY00000000

a Estimated by dividing the total number of reads generated (in base pairs) by the genome size of about 4.1 Mb (4,100,000 bp).

b *N*_50_, the minimum contig length needed to cover 50% of the genome, indicating that half of the genome sequence is in contigs larger than or equal to the *N*_50_ contig size.

**TABLE 3:** Properties of strains belonging to GC1, lineage 2

Isolate^*a*^	Date	Country	Source	ST^*b*^		KL	OCL	RI in *comM*^*c*^	Presence of:								Amino acid in^*d*^:		GenBank accession no.
				IP	OX				IS*Aba1*- *ampC*^*e*^	*oxa*^*f*^	*cmlA1*	*aphA*^*g*^	*mer*	*sul2*	*aadB*	*tet*^*h*^	GyrA81	ParC84	
TG19582	nk^*i*^	nk	nk	1	231	1	1	Intact	− (1)	−	−	−	−	+	−	−	L	S	AMIV00000000
**PR332**	nk	USA	nk	81	498	12	2	Intact	+ (17)	−	−	1a	+	+	+	−	L	L	NGDV01000000
OIFC074	2003	USA	nk	19	231	1	5	Tn*6022*	+ (3)	−	+	1b	−	+	−	*A*(B)	L	S	AMDE01000000
Naval-21	2006	USA	Wound	19	946	15	1	Tn*6022*	+ (3)	−	+	1b	−	+	−	*A*(B)	L	L	AMSY01000000
6013150	2007	UK	Skin	81	498	12	2	Intact	+ (17)	−	−	1a	+	+	+	−	L	L	ACYQ00000000
6013113	2007	UK	Skin	81	372	12	2	Intact	+ (17)	−	−	1a	+	+	+	−	L	L	ACYR00000000
D36	2008	Australia	Wound	81	498	12	2	AbaR4	+ (17)	23	−	1a	+	+	+	−	L	L	CP012952^*j*^
MRSN 3527	2011	USA	Wound	81	498	12	2	AbaR4	+ (17)	23	−	6	+	+	+	−	L	L	JPHZ00000000
**ABS029**	**2012**	Iran	Wound	328	1972	18	2	AbaR4	+ (80)	23, 24	−	6	+	+	−	39	L	L	WIOH00000000
**ABS042**	**2012**	Iran	Wound	328	498	13	2a	AbaR4	+ (81)	23, 24	−	6	−	+	+	−	L	L	WIOG00000000
**ABS062**	**2012**	Iran	Wound	328	1972	18	2	AbaR4	+ (80)	23, 24	−	6	+	+	−	39	L	L	WIOF00000000
**ABS063**	**2012**	Iran	Wound	328	1972	18	2	AbaR4	+ (80)	23, 24	−	6	+	+	−	39	L	L	WIOE00000000
**ABS078**	**2013**	Iran	Wound	328	498	13	2a	AbaR4	+ (81)	23, 24	−	6	−	+	+	−	L	L	WIOD00000000
**ABS103**	**2013**	Iran	Wound	328	498	13	2a	AbaR4	+ (81)	23, 24	−	6	+	+	+	−	L	L	WIOC00000000
**ABS104**	**2013**	Iran	Blood	328	498	13	2a	AbaR4	+ (81)	23, 24	−	6	+	+	+	−	L	L	WIOB00000000
**ABS122**	**2013**	Iran	Wound	328	1972	18	2	AbaR4	+ (80)	23, 24	−	6	+	+	−	39	L	L	WIOA00000000
**ABS201**	**2013**	Iran	Wound	328	1972	18	2	AbaR4	+ (80)	23, 24	−	6	+	+	−	39	L	L	VJZY00000000

a Genomes sequenced or analyzed in this study are shown in bold. Analysis of the remaining genomes from reference 10 is shown for ease of comparison.

b IP, Institut Pasteur scheme, which uses the *cpn60*, *fusA*, *gltA*, *pyrG*, *recA*, *rplB*, and *rpoB* genes; OX, Institut Oxford scheme, which uses the *cpn60*, *gltA*, *gyrB*, *gdhB*, *recA*, *cpn60*, and *rpoD* genes.

c RI, the resistance island found in the chromosomal *comM* gene. In lineage 1, this gene is often occupied by the AbaR0/3-type RI, while it is either intact or interrupted by AbaR4 or Tn*6022*.

d Leucine (L) and serine (S) at positions of 81 and 84 of the GyrA and ParC proteins. Fluoroquinolone-resistant strains often include an L at these positions, and sensitive strains tend to include S.

e Numbers in parentheses indicate the *ampC* allele numbers. All allele numbers are those used in the *ampC* database, publicly available at https://pubmlst.org/abaumannii/.

f 23, 24: the *oxa23* and *oxa24* carbapenem resistance genes, respectively.

g 1a, 1b, and 6, *aphA1a*, *aphA1b*, and *aphA6*, respectively.

h *A*(B) and 39, the *tetA*(B) or *tet39* tetracycline resistance gene, respectively.

i nk, not known.

j A complete genome is available for D36 (31). D36 carries 4 plasmids (GenBank accession numbers CP012953 to CP012956).

10.1128/mSphere.00164-20.2 TABLE S2 Single nucleotide differences between GC1 strains belonging to lineage 1 and lineage 2. Download Table S2, CSV file, 0.1 MB. Copyright © 2020 Douraghi et al. 2020 Douraghi et al. This content is distributed under the terms of the Creative Commons Attribution 4.0 International license.

Further, analysis of the recombination patches across the entire chromosomes showed that the Iranian strains share several recombinant regions with other lineage 2 strains, while they also include few recombination blocks specific to their branch (Fig. 2). The Iranian strains were placed on three branches, and interestingly, each branch contained novel (branch-specific) recombination patches. These analyses confirmed the assignment of the Iranian strain to lineage 2 of GC1 and indicated their continued evolution and genetic exchange via homologous recombination.

**(ii) Surface polysaccharide loci.** Capsular polysaccharide (CPS) is a major virulence factor for A. baumannii, and the genes at the K locus (KL), which directs its synthesis, have previously been shown to vary between isolates of the GC1 clone (10). In that study, we demonstrated that strain D36 and the related strains 6013113 and 6013150 carry the KL12 CPS biosynthesis gene cluster (10). Here, KL12 was found in one more isolate, PR332, belonging to ST81 (Table 3). However, the Iranian strains were found to carry either KL13 or KL18, with the KL type separating the two distinct phylogenetic subclades in which the Iranian strains are positioned (Fig. 2 and Table 3). The genomes of other lineage 2 isolates that do not belong to the ST81/ST328 subclade were shown to carry either KL1 or KL15 (10).

**FIG 3:**
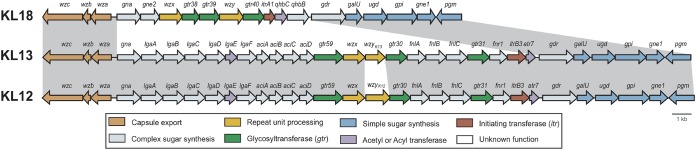
Arrangement of the CPS biosynthesis gene clusters at the K locus in isolates belonging to the ST81/ST328 subclade of lineage 2. Genes are colored according to the functions of their predicted products, and the scheme is shown at the bottom. The figure is drawn to scale from representative KL sequences available as GenBank accession numbers MF522811 (KL18), MF522810.1 (KL13), and JN107991.2 (KL12). Shading between gene clusters indicates regions of nucleotide sequence similarity.

The arrangements of the KL13 and KL18 gene clusters (Fig. 3) have been described previously (34, 35), though neither has been reported in a GC1 isolate to date. Interestingly, the KL13 gene cluster is closely related to KL12, and both include genes for the synthesis of the rare non-2-ulosonic acid sugar 5,7-di-*N*-acetylacinetaminic acid, also known as Aci5Ac7Ac (34). The two genetic arrangements differ only in a small segment that includes a single gene (Fig. 3). However, this small replacement leads to a structural change in the K12 and K13 CPS that are produced (34). Similarly, the KL18 gene cluster is closely related to KL17, which has so far been reported in only a single GC1 lineage 1 isolate, isolate G7 (10). However, recently, it has been argued that the differences between the KL17 and KL18 gene clusters are unlikely to result in a structural change in the CPS (35).

In addition to the K locus, a second chromosomal region involved in the synthesis of the outer core (OC) component of the lipooligosaccharide (known as the OC locus [OCL]) (36) was also found to vary in our previous analysis of GC1 (10). Isolates in the ST81 clade were previously shown to carry OCL2 (10), and the OCL2 arrangement was identified in the additional isolates belonging to the ST81/ST328 subclade, though OCL2 in the Iranian isolates with KL13 had an IS*Aba12* interrupting the *gtrOC9* gene, and this variant was designated OCL2a.

**(iii) Sequence types.** We previously showed that, despite belonging to the same lineage within GC1, lineage 2 strains belong to several sequence types of ST_IP_ and the Oxford MLST scheme (ST_OX_) (10). Using PCR and sequencing, we showed that all Iranian strains belong to ST328_IP,_ while D36 and the 4 other strains in D36 clade belong to ST81 (a *pyrG* SLV of ST328; see above), indicating an ST81/ST328-specific clade within lineage 2. Analysis of the Oxford sequence types indicated that the strains also exhibited a higher degree of diversity. These differences were mainly due to the presence of a different *gpi* allele, as previously shown (10). This gene is located within the K locus for capsular polysaccharide (CPS) biosynthesis (36), and the differences showed structural diversity on the cell surface (Table 3). This corresponds to the difference in ST_OX_, as KL13 isolates belonged to ST498 and KL18 isolates belonged to ST1972 (Table 3). The K locus represents the tallest peak in the SND density plot shown in Fig. 2, indicating extensive recombination events in this region.

**(iv) Resistance to 3rd-generation cephalosporins and acquisition of the *ampC* gene.** Resistance to 3rd-generation cephalosporins may be conferred by the insertion of IS*Aba1* or IS*Aba125* upstream of the *ampC* gene, which enhances expression (37). We previously showed that IS*Aba1* is present in the same location in all other lineage 2 strains studied before, except strain TG19582 (10) (Table 3). Here, consistent with the ceftazidime and cefotaxime resistance profiles of the Iranian strains and the results of PCR analysis (see above), IS*Aba1* was found to be 9 bp away from the start of the chromosomal *ampC* gene in all 9 sequenced Iranian strains.

**FIG 4:**
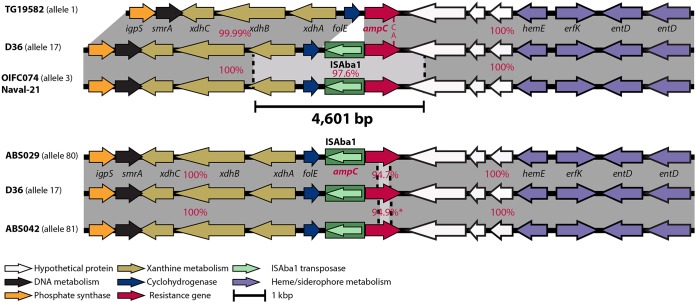
Alignment of the chromosomal *ampC* gene and its surrounding regions (10 kbp on either side). Horizontal arrows indicate the directions and orientations of the genes, and the green filled box indicates IS*Aba1*, with the arrow inside indicating the direction of the transposase gene. Genes are color coded based on their function, and the key is shown at the bottom. Shades of gray indicate regions with significant identity, and red numbers indicate their percent DNA sequence identities to the D36 genome.

We also previously showed that GC1 strains can gain IS*Aba125*- and IS*Aba1*-activated *ampC* genes, along with a surrounding segment of the chromosome, by horizontal transfer from an exogenous source (37, 38). Hence, we explored the surrounding regions of the *ampC* gene in all lineage 2 strains for evidence of whether different *ampC* alleles were the result of horizontal transfer events. Analysis of the 1,152-bp *ampC* gene in the 9 Iranian strains revealed two potential internal recombination patches of about 550 bp with ∼94% DNA identity compared to the sequences of their corresponding regions in strains D36 and TG19582 (Fig. 4). However, we could not identify the source of these patches in GenBank or WGS database searches. The new alleles found here in Iranian strains, *ampC80* and *ampC81*, have been deposited in the *ampC* database, publicly available at http://pubmlst.org/abaumannii/. All other *ampC* allele numbers in Table 3 are also those used in the *ampC* database.

We previously showed that strains OIFC074 and Naval-21 contain a different *ampC* allele (10). Here, analysis of *ampC* and its surrounding sequence indicated a 4.6-kb recombination patch in OIFC074 and Naval-21, which shared 97.6% DNA identity with the corresponding region in D36 and TG19582 (Fig. 4), indicating that a short segment, including an IS*Aba1*-activated *ampC* gene, has been horizontally transferred and incorporated into these two genomes. However, searches of the GenBank and draft genomes in WGS databases did not result in identification of the source for this *ampC* recombinant region.

**(v) Resistance to fluoroquinolones.**All Iranian strains contained the same *gyrA* and *parC* alleles as strain D36, encoding a leucine (L) at positions 81 and 84 of the GyrA and ParC proteins, respectively, which is consistent with their nalidixic acid and ciprofloxacin resistance phenotype (Table S1 and Table 3). The remaining lineage 2 strains also had the same *gyrA* and *parC* gene alleles as D36 (GenBank accession no. CP012952) (31).

**FIG 5:**
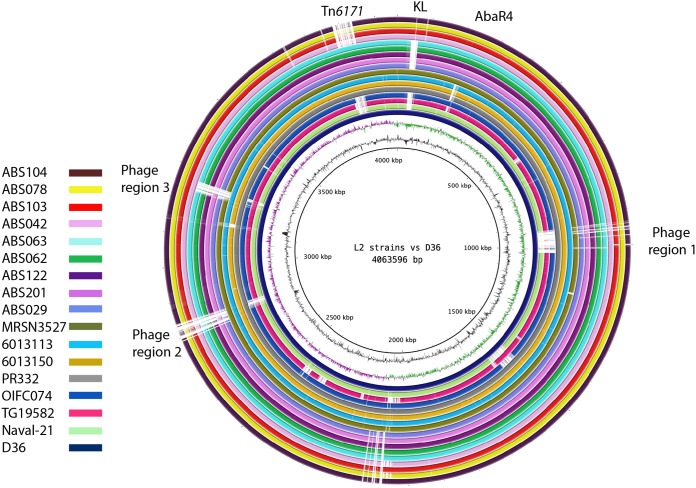
Comparison of the chromosomes of the lineage 2 strains using the BLAST Ring Image Generator (BRIG). The chromosomal sequences of all lineage 2 (L2) strains were compared to the sequence of the chromosome of D36, and the innermost ring indicates D36, which was also used as a control. The second ring illustrates the GC content, followed by the GC skew. The fourth ring (in dark blue) also indicates D36, which was also used as a control. The chromosomes of other lineage 2 strains are color coded, with the key being given on the left.

**(vi) Prophage genomes.** Three intact prophage genomes of 36.3 kb, 36.4 kb, and 95.9 kb were previously identified in strain D36 (31). Here, screening of the Iranian strains indicated the presence of a variant of prophage region 1 in all strains, while prophage region 2 was missing. Interestingly, all KL13 strains also contained prophage region 3, while KL18 strains lacked this region (Fig. 5). All ST81 strains included all three prophage regions. Analysis of the other lineage 2 genomes indicated that the majority had either complete copies of all three prophage genomes or a remnant of them. In the first subclade, which included strains TG19582, Naval-21, and OIFC074, large portions of all 3 prophage regions appeared to be missing, with only very small portions remaining (Fig. 5), indicating a complex history. This was not investigated further.

**(vii) Antibiotic resistance genes in plasmids.** Analysis of resistance genes in Iranian strains and other lineage 2 strains (Table 3) confirmed that, as previously shown (10), strains in lineage 2 included a set of antibiotic resistance genes completely different from those in the bulk of GC1 strains belonging to lineage 1 (10). Moreover, the 9 sequenced Iranian strains differed from other lineage 2 strains and other members of the ST81/ST328 clade by the presence of an *oxa24* carbapenem resistance gene, in addition to *oxa23* (Table 3). The *oxa24* gene, encoding the OXA-72 variant, was found within a p*dif* module and was identical to the *oxa24* modules previously described (39, 40). The *oxa24* p*dif* module was found in an ∼15-kb contig, which appears to represent a plasmid, as it encodes a putative replication initiation protein (Rep) that is 98.9% identical to that encoded by the A. baumannii plasmid pMAC (GenBank accession no. AY541809.1).

Among the strains sequenced, the five tetracycline-resistant isolates that contained *tet39* belonged to the same KL18 subclade in the recombination-free phylogeny (Fig. 2). The *tet39* gene was found in an approximately 4-kb contig within a p*dif* module identical to that described previously (41). This contig also encodes a putative plasmid replication initiation protein (Rep) that is 98% identical to the one in A. baumannii RCH52 (GenBank accession no. KT346360) (42), suggesting the presence of a similar plasmid in the Iranian strains. However, the complete plasmid sequence could not be assembled due to the presence of several repeated sequences. Here, the *tet39* tetracycline resistance gene was shown to be present in all 33 tetracycline-resistant Iranian strains (Table 3) and to account for their tetracycline resistance phenotype (see the PCR screening results above). To the best of our knowledge, this is the first time that *tet39* has been seen in lineage 2 of GC1.

Interestingly, the *sul2* sulfonamide resistance gene was carried by all GC1 lineage 2 isolates, while this gene has so far rarely been seen in strains belonging to lineage 1, which carry *sul1* embedded within the AbaR0/3-type resistance islands as part of the 3′ conserved sequence segment of the class 1 integron (23). Sequence analysis showed that in the Iranian isolates the *sul2* gene is located in a plasmid (Table 4), similar to pD36-4 previously reported in D36 (43).

**TABLE 4:** Plasmid content of strains belonging to GC1, lineage 2, ST81/ST328 clade

Isolate	Presence of the following plasmid:						
	pD36-1	pD36-2 (pRAY*)	pD36-3	pD36-4	p*oxa24*	p*tet39*	pTn*aphA6*
D36	+	+	+	+	−	−	−
6013150	−	+	+^*a*^	+	−	−	−
6013113	−	+	+^*a*^	+	−	−	−
MRSN 3527	+^*b*^	+	+^*c*^	+^*d*^	−	−	−
PR332	−	+	+	+	−	−	−
ABS029	+	−	+	+	+	+	+
ABS042	+	+	+	+^*e*^	+	−	+
ABS062	+	−	+	+	+	+	+
ABS063	+	−	+	+	+	+	+
ABS078	+	+	+	+^*e*^	+	−	+
ABS103	+	+	+	+	+	−	+
ABS104	+	+	+	+	+	−	+
ABS122	+	−	+	+	+	+	+
ABS201	+	−	+	+	+	+	+

a Includes indels of ∼0.3 kbp and 1.3 kb.

b A total of 438 bp is missing.

c Containing only 2 fragments (0.7 and 2.2 kb) of pD36-3.

d The Tn*4352*::IS*Aba1* structure is missing, likely due to an IS*26*-mediated deletion event.

e The *mer* module is missing.

Using PCR, a 100% correlation between the presence of the *aadB* gene in the pRAY context and the tobramycin, gentamicin, and kanamycin resistance phenotype was shown (Table 1). Here, the presence of pRAY* was confirmed in 4 genomes (Tables 3 and 4). Interestingly, all these 4 genomes contained KL13 (Table 3).

**FIG 6:**
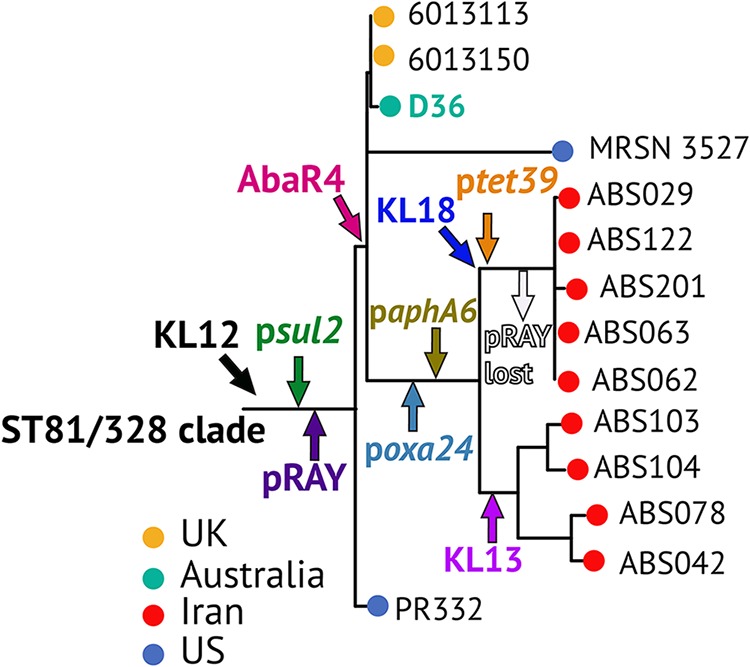
Schematic representation of events (antibiotic resistance gene acquisition and capsule switching) in the ST81/ST328 clade. Arrows show specific branch points where each resistance gene or KL was acquired.

We recently showed that amikacin resistance is due to the presence of the *aphA6* amikacin resistance gene, located in Tn*aphA6*, in a large set of GC1 Iranian strains, including the ones analyzed here (28). Tn*aphA6* is made up of a central segment containing *aphA6* flanked by two copies of IS*Aba125*. To locate Tn*aphA6*, here, the 9 sequenced genomes were searched for contigs with IS*Aba125* at their ends. In each genome, 9 contigs were found; 7 contigs had the IS*Aba125* sequence at one end, 1 contig which also had the *apha6* sequence contained the IS*Aba125* sequence at both ends, and an additional contig contained an internal sequence of IS*Aba125*. All of these contigs had a coverage of >15-fold relative to contigs containing chromosomal genes, indicating that all IS*Aba125* copies and, hence, Tn*aphA6* must be located on a plasmid estimated to be 15 to 20 kb. A more detailed context of the plasmids was not pursued further. However, the analysis performed here indicates that, except for *oxa23*, which is located in AbaR4 in the chromosome, all other resistance genes appear to have been acquired via 5 different plasmids (Table 4; Fig. 6).

### (viii) Cryptic plasmids.

In addition to two plasmids that carry antibiotic resistance genes (plasmids pD36-2 and pD36-4), we previously showed that the lineage 2 strain D36 contains two cryptic plasmids (plasmids pD36-1 and pD36-3) (43). We also previously screened other members of lineage 2 to find them (43). Here, the cryptic pD36-3 plasmid was found in all strains belonging to the ST81/ST328 subclade (Table 4), while the pD36-1 plasmid was present only in D36 and the Iranian strains (Table 4).

## DISCUSSION

CRAB was placed as the number 1 critical priority pathogen on the World Health Organization’s list of 12 multiresistant bacteria for which immediate research on new therapeutics and antibiotic development is required (44). However, despite the global distribution of CRAB and the need to have data from all countries, there is a substantial gap in WGS data from most geographical regions across the globe, as the bulk of the current publicly available genome sequence data is from only 4 countries, namely, the United States, China, Thailand, and Australia (19). The lack of sequence data has made it difficult to track the spread of resistance at the global level and determine the population structure of this microorganism (19).

Over the last decade, a large number of studies have reported the alarming rate of carbapenem resistance among A. baumannii isolates in Iran (14–16). However, virtually all these studies have included only the phenotypic determination of carbapenem resistance and PCR screening for carbapenem resistance genes, combined with very limited phylogenetic analysis using traditional methods (14–16), making it impossible to place strains recovered from this region in the global context. Here, for the first time, we investigated the phylogenetic relationships among a set of 9 strains, representing 50 CRAB Iranian isolates that caused several outbreaks and that were recovered in 2012 and 2013 from a single hospital in Tehran, Iran, in the context of isolates belonging to the same lineage recovered in regions around the world. This study showed that all Iranian outbreak strains belong to the same sequence type, ST328 in the Institut Pasteur scheme, and share many properties, such as resistance to the frontline carbapenem antibiotics, conferred by the carbapenem resistance *oxa23* gene in Tn*2006* within AbaR4, which is located in the chromosomal *comM* gene, and a plasmid that carries *oxa24*. This study further represents the first analysis of the genetic context of the most widespread carbapenem resistance gene, *oxa23* (19), in A. baumannii isolates recovered from Iran. In A. baumannii strains that belong to the two major global clones, resistance genes often reside in the chromosome within large genomic islands (6, 23, 24). However, the only chromosomal resistance gene found in Iranian strains is *oxa23*, and all other resistance genes, *aadB*, *aphA6*, *oxa24*, *tet39*, and *sul2*, have been acquired by 5 different plasmids (Fig. 6). This highlights the role of plasmids in the acquisition of these important resistance determinants in this subset of lineage 2 strains.

Though we showed that the Iranian isolates studied here fall into the second defined lineage of GC1, it is noteworthy that the Iranian strains differed from each other in many ways. Differences included the presence of different recombination patches across their genomes, different plasmid and prophage contents, and different *ampC* alleles, as well as the carriage of different OC and K loci. The different K loci explain their assignment to different sequence types in the Oxford MLST scheme. Interestingly, each of KL13 and KL18 was correlated with certain properties, as all strains with KL13 contained OCL2a, *ampC81*, pRAY*, and phage region 3, while strains with KL18 contained OCL2, *tet39*, *ampC80*, and prophage regions 1 and 2. These differences could suggest a single entry into the hospital environment, followed by continued evolution, leading to the separation of the KL13 and KL18 subclades over 2012 and 2013. This raises concerns about the hospital’s colonization with CRAB and the need to review infection control measures regularly.

Previously, it was shown that the most recent common ancestor of GC1 arose in about 1960, and subsequently, in about 1967, members of GC1 diverged into two phylogenetically distinct lineages (10). All strains belonging to lineage 1 included either an AbaR-type resistance island or a remnant of it in the *comM* gene, whereas lineage 2 strains, including D36, either contained an intact *comM* or carried Tn*6022* or AbaR4 in the *comM* gene (10). This study further demonstrates that the multidrug-resistant ST81/ST328 subclade of lineage 2 within global clone 1 has clearly diverged from a common precursor and from the bulk of GC1 isolates and is defined by the acquisition of the AbaR4 resistance island and IS*Aba1* upstream of the *ampC* gene. These genetic features confer the ability to resist broad-spectrum β-lactam antibiotics, including 3rd-generation cephalosporins and the frontline antibiotics, the carbapenems.

This study provides clear evidence that strains belonging to lineage 2 have undergone different evolutionary changes to achieve their resistance phenotypes, evidenced by the presence of a resistance gene complement completely different from that of lineage 1 strains, and that all but *oxa23* have been acquired by a different plasmid. This, in many ways, indicates the versatility of A. baumannii genomes and the wide range of antibiotic resistance genes and mobile genetic elements that could be involved with antibiotic resistance in this microorganism. It also indicates that all these plasmids must be compatible.

Given that D36 is a military isolate, it was previously hypothesized that GC1 isolates and, in particular, strains belonging to lineage 2 might have originated from the Middle East (10), and evidence was provided to support the suggestion that D36 might be a carbapenem-resistant strain that was introduced into an Australian hospital by a member of the military (30). This study demonstrates a close relationship of the Iranian outbreak isolates recovered from hospital H5 in Iran with the Australian military isolate D36 and showed that the Iranian outbreak isolates belong to the same lineage as D36. These findings, combined with finding a single ST328 strain in the MLST database, provide further evidence that the Middle East region might be a reservoir for GC1 strains that belong to lineage 2. However, determining a more accurate distribution rate of lineage 2 strains in the Middle East and also globally warrants further investigation and more genome sequence data.

## MATERIALS AND METHODS

### Bacterial strains.

A total of 50 isolates resistant to carbapenems were recovered from several outbreaks in a single Tehran hospital (hospital H5) between 2012 and 2013 and identified as GC1 (22) and were further examined in this study. Their features are listed in Table 1.

### Antimicrobial susceptibility testing.

In addition to the profiles of resistance to 20 antibiotics previously reported (22), the profiles of resistance to an additional 8 antibiotics, including ampicillin (25 μg), kanamycin (30 μg), neomycin (30 μg), nalidixic acid (30 μg), netilmicin (30 μg), streptomycin (25 μg), spectinomycin (25 μg), and sulfonamide (100 μg), were determined using the standard Kirby-Bauer disk diffusion method as described elsewhere (30). Strains were classified as resistant and susceptible according to the Clinical and Laboratory Standards Institute (CLSI) guidelines for Acinetobacter spp. (45) and calibrated dichotomous sensitivity disk diffusion assay (CDS) (http://cdstest.net/) when a CLSI breakpoint for Acinetobacter spp. was not available (for netilmicin, streptomycin, spectinomycin, sulfamethoxazole, nalidixic acid, and rifampin).

### PCR amplification, DNA sequencing, and sequence analysis.

PCR amplification was carried out using published primers for various antibiotic resistance genes, including *aphA1*, *tetA*(A), *sul1*, *bla*_TEM_, and *catA1* (5, 6, 29); the *comM* gene; features of the AbaR0/3-type islands (AbaR-type J1 to J6 junctions, *intI1*, *top*, and Tn*6018*-L and Tn*6018*-R) (6); and *aadB* in pRAY (29). Published primers and conditions were also used to identify the carbapenemase resistance genes *oxa23*, *oxa24*, and *oxa58* (46) and to detect IS*Aba1* upstream of the chromosomal *ampC* gene (47). The intrinsic *oxa-Ab* gene (also referred to as *bla*_OXA-51_ elsewhere) was also amplified and sequenced for representative strains using primers previously published (21). For primers and amplicons up to 3 kb, reaction and cycling conditions were as described elsewhere (30). For larger amplicons, Phusion DNA polymerase (New England Biolabs) and Phusion HF buffer replaced *Taq* polymerase and PCR buffer. Cycling conditions included an initial denaturation cycle at 98°C for 30 s, followed by 35 cycles of denaturation at 98°C for 10 s, annealing at 60°C for 30 s, and extension at 72°C for 30 s per 1 kbp of expected PCR product. The final extension was at 72°C for 10 min. The PCR amplicons were separated using standard 1% agarose gels, stained with ethidium bromide, and visualized as described elsewhere. Sequencing was performed as described previously (30).

### Whole-genome sequencing, genome assembly, and *in silico* screening of genomes.

Genomic DNA isolated from 9 representative strains (identified in boldface in Table 1), including 1 strain from each hospital ward isolated in 2012 and 1 representative strain from each hospital ward isolated in 2013 (in total, 2 strains from each ward), were sequenced in-house at the University of Technology Sydney, using Illumina MiSeq technology. Whole-genome sequence data were obtained, and paired-end reads of 250 bp were assembled *de novo* using the SPAdes algorithm (48).

Antibiotic resistance genes and the contigs carrying them were identified in all genomes, as well as in all lineage 2 sequences found in GenBank, using the ResFinder program (https://cge.cbs.dtu.dk/services/ResFinder/). A local database was created and used to screen for specific genomic regions using the NCBI standalone software BLAST (ftp://ftp.ncbi.nlm.nih.gov/blast/executables/blast+/LATEST/). Prophage genomes were found using searches of the PHASTER database (https://phaster.ca/) (49). Plasmid content was examined using the full sequence of plasmids previously found in strain D36, which was used as a representative of lineage 2 strains. BLAST results with >90% coverage and >95% DNA identity were considered positive and, hence, indicated that a given plasmid was present.

### Phylogenetic analysis.

To determine the locations of the 9 Iranian outbreak isolates in a GC1 phylogenetic tree, a maximum likelihood tree was constructed from the core genome alignment as previously described (10). The core genome alignment also included the sequences of a number of GC1 isolates known to belong to either lineage 1 or lineage 2, which were used as controls (10). In addition to strains previously shown to be members of lineage 2, two isolates (PR322 and MRSN 3527) that were identified as ST81 (a known ST in lineage 2) in the GenBank Whole-Genome Shotgun database were also included in the phylogenetic analysis. To draw a whole-genome phylogenetic tree, Illumina sequence reads for all isolates were mapped to the A1 GC1 reference genome (GenBank accession no. CP010781) (33), using the snplord pipeline, available at https://github.com/CJREID/snplord. The snplord pipeline uses the Snippy tool (available at https://github.com/tseemann/snippy) to generate a whole-genome alignment. Briefly, Snippy mapped all reads to the reference genome using the bwa (v0.7.12) and minimap2 (v2.0) programs and default parameters. High-quality variant sites were called using SAMtools (v1.3.1.24) with standard quality filtering, as described previously (10). Single nucleotide differences (SNDs) in recombinant regions were identified and removed using the Gubbins (v2.1.025) program (50) with default parameters, including a default taxa filtering percentage of 25%. A maximum likelihood phylogenetic tree was inferred from the resulting recombination-filtered alignment using the RAxML (v.8) program with the GAMMA model. The tree was visualized and annotated using the R package ggtree (v1.12.027). Recombination blocks were plotted against the phylogenetic tree in R (v3.5.2) using the ggtree (v1.16.6) and ggplot2 (v3.2.1) packages and the PlotTree program, available at https://github.com/katholt/plotTree. Bootstrap values were calculated using 10 independent runs of RAxML with 1,000 bootstraps, which each gave nearly identical results.

### MLST.

The Institut Pasteur (IP) multilocus sequence typing (MLST) scheme, which uses 7 housekeeping genes (*cpn60*, *fusA*, *gltA*, *pyrG*, *recA*, *rplB*, and *rpoB*), was used to determine the sequence type of all 50 strains using the primers and conditions specified previously (32). An ST number was assigned by comparing the allele sequences to the ones on the MLST site (http://pubmlst.org/abaumannii/). The Oxford and Institut Pasteur multilocus sequence types were also determined *in silico* for the strains sequenced here.

### Capsule and lipooligosaccharide biosynthesis genes.

Genetic arrangements located at the K locus (KL) for capsular polysaccharide (CPS) biosynthesis and the OC locus (OCL) for synthesis of the outer core component of the lipooligosaccharide (LOS) were determined using the curated A. baumannii KL and OCL sequence databases available through Kaptive (51).

### Data availability.

Draft genome sequences of strains ABS029, ABS042, ABS062, ABD063, ABS078, ABS103, ABS104, ABS122, and ABS201 have been deposited in the GenBank/EMBL/DDBJ database and are publicly available under accession numbers WIOH00000000, WIOG00000000, WIOF00000000, WIOE00000000, WIOD00000000, WIOC00000000, WIOB00000000, WIOA00000000, and VJZY00000000, respectively. The new *ampC* alleles found here in Iranian strains, *ampC80* and *ampC81*, have been deposited in the *ampC* database, publicly available under http://pubmlst.org/abaumannii/.
