# Supplementary material for: Accumulation of Antibiotic Resistance Genes in Carbapenem-Resistant Acinetobacter baumannii Isolates Belonging to Lineage 2, Global Clone 1, from Outbreaks in 2012–2013 at a Tehran Burns Hospital
Source: mSphere. 2020 Apr 8;5(2):e00164-20. doi: 10.1128/mSphere.00164-20 (PMC7142300; doi:10.1128/mSphere.00164-20)
Supplement: TABLE S1 [file mSphere.00164-20-st001.docx]

TABLE S1* Antibiotic resistance profiles of GC1 strains.

| **Isolate** | **Ap** | **Sm** | **Sp** | **Su** | **Tc** | **Tp** | **Km** | **Nm** | **CTX** | **CAZ** | **Gm** | **Cip** | **AK** | **Nx** | **Tm** | **Ne** | **Ipm** | **Mem** | **TIM** | **Rif** | **SAM** | **FEP** | **DOR** | **TZP** | **CRO** | | **MIN** | | **DOX** | **LVX** |
| --- | --- | --- | --- | --- | --- | --- | --- | --- | --- | --- | --- | --- | --- | --- | --- | --- | --- | --- | --- | --- | --- | --- | --- | --- | --- | --- | --- | --- | --- | --- |
| ABS249 | 7 | 7 | 7 | 7 | 10 | 7 | 7 | 7 | 7 | 7 | 7 | 7 | 7 | 7 | 7 | 14 | 7 | 7 | 7 | 16 | 13 | 7 | 7 | 9 | 7 | 18 | | 19 | | 11 |
| ABS260 | 7 | 7 | 7 | 7 | 12 | 7 | 7 | 9 | 7 | 7 | 7 | 7 | 13 | 7 | 7 | 15 | 7 | 7 | 7 | 16 | 7 | 7 | 7 | 12 | 8 | 16 | | 16 | | 8 |
| ABS267 | 7 | 7 | 7 | 7 | 7 | 7 | 7 | 8 | 7 | 7 | 14 | 7 | 8 | 7 | 19 | 15 | 7 | 7 | 7 | 17 | 17 | 7 | 7 | 10 | 7 | 15 | | 10 | | 9 |
| ABS288 | 7 | 7 | 7 | 7 | 7 | 7 | 7 | 7 | 7 | 7 | 7 | 12 | 7 | 7 | 7 | 14 | 9 | 7 | 7 | 7 | 7 | 7 | 7 | 8 | 7 | 20 | | 12 | | 10 |
| ABS290 | 7 | 7 | 7 | 7 | 7 | 7 | 7 | 7 | 7 | 7 | 7 | 7 | 7 | 7 | 7 | 13 | 12 | 8 | 7 | 16 | 12 | 7 | 7 | 8 | 7 | 13 | | 9 | | 11 |
| ABS178 | 7 | 7 | 12 | 7 | 14 | 7 | 7 | 12 | 7 | 7 | 7 | 7 | 11 | 7 | 7 | 19 | 7 | 7 | 7 | 15 | 7 | 7 | 7 | 9 | 7 | 14 | | 20 | | 13 |
| ABS101 | 7 | 7 | 7 | 7 | 7 | 7 | 7 | 8 | 7 | 7 | 16 | 7 | 10 | 7 | 20 | 14 | 7 | 7 | 7 | 15 | 16 | 7 | 7 | 10 | 7 | 15 | | 11 | | 10 |
| ABS103 | 7 | 7 | 7 | 7 | 12 | 7 | 7 | 7 | 7 | 7 | 7 | 7 | 7 | 7 | 7 | 14 | 7 | 7 | 7 | 15 | 16 | 7 | 7 | 10 | 7 | 17 | | 16 | | 9 |
| ABS105 | 7 | 7 | 7 | 7 | 13 | 7 | 7 | 10 | 7 | 7 | 7 | 7 | 10 | 7 | 7 | 14 | 7 | 7 | 7 | 15 | 15 | 7 | 7 | 8 | 7 | 17 | | 7 | | 12 |
| ABS121 | 7 | 7 | 7 | 7 | 7 | 7 | 7 | 7 | 7 | 7 | 16 | 7 | 8 | 7 | 19 | 14 | 7 | 7 | 7 | 15 | 15 | 7 | 7 | 7 | 7 | 16 | | 12 | | 10 |
| ABS104 | 7 | 7 | 15 | 7 | 12 | 7 | 7 | 12 | 7 | 7 | 7 | 7 | 10 | 7 | 15 | 20 | 7 | 7 | 7 | 15 | 17 | 7 | 7 | 9 | 7 | 18 | | 18 | | 13 |
| ABS083 | 7 | 7 | 8 | 7 | 11 | 7 | 7 | 12 | 7 | 7 | 7 | 7 | 14 | 7 | 7 | 15 | 7 | 7 | 7 | 16 | 16 | 7 | 7 | 11 | 7 | 22 | | 11 | | 7 |
| ABS063 | 7 | 7 | 13 | 7 | 7 | 7 | 7 | 13 | 7 | 7 | 21 | 7 | 13 | 7 | 21 | 18 | 7 | 7 | 7 | 15 | 17 | 7 | 7 | 7 | 7 | 17 | | 9 | | 13 |
| ABS219 | 7 | 7 | 7 | 7 | 13 | 7 | 7 | 7 | 7 | 7 | 16 | 7 | 9 | 7 | 18 | 14 | 8 | 7 | 7 | 16 | 16 | 7 | 7 | 12 | 7 | 20 | | 21 | | 10 |
| ABS283 | 7 | 7 | 8 | 7 | 7 | 7 | 7 | 10 | 7 | 7 | 7 | 7 | 10 | 7 | 7 | 15 | 7 | 7 | 7 | 15 | 15 | 7 | 7 | 7 | 7 | 16 | | 7 | | 9 |
| ABS085 | 7 | 7 | 7 | 7 | 7 | 7 | 7 | 10 | 7 | 7 | 7 | 7 | 13 | 7 | 7 | 14 | 7 | 7 | 7 | 15 | 16 | 7 | 7 | 7 | 7 | 22 | | 12 | | 9 |
| ABS094 | 7 | 7 | 7 | 7 | 13 | 7 | 7 | 8 | 7 | 7 | 7 | 7 | 7 | 7 | 7 | 15 | 7 | 7 | 7 | 15 | 11 | 7 | 7 | 12 | 9 | 16 | | 15 | | 7 |
| ABS062 | 7 | 7 | 13 | 7 | 7 | 7 | 7 | 12 | 7 | 7 | 21 | 7 | 14 | 7 | 20 | 18 | 7 | 7 | 7 | 16 | 15 | 7 | 7 | 9 | 7 | 18 | | 10 | | 7 |
| ABS029 | 7 | 7 | 7 | 7 | 7 | 7 | 7 | 7 | 7 | 7 | 16 | 7 | 10 | 7 | 20 | 13 | 7 | 7 | 7 | 14 | 15 | 7 | 7 | 7 | 7 | 15 | | 7 | | 10 |
| ABS035 | 7 | 7 | 7 | 7 | 7 | 7 | 7 | 8 | 7 | 7 | 7 | 7 | 15 | 7 | 7 | 13 | 7 | 7 | 7 | 15 | 13 | 7 | 7 | 10 | 7 | 20 | | 12 | | 9 |
| ABS042 | 7 | 7 | 7 | 7 | 7 | 7 | 7 | 8 | 7 | 7 | 7 | 7 | 12 | 7 | 7 | 13 | 7 | 7 | 7 | 17 | 14 | 7 | 7 | 7 | 7 | 18 | | 7 | | 9 |
| ABS045 | 7 | 7 | 7 | 7 | 7 | 7 | 7 | 8 | 7 | 7 | 16 | 7 | 9 | 7 | 18 | 14 | 7 | 7 | 7 | 15 | 16 | 7 | 7 | 7 | 7 | 14 | | 7 | | 10 |
| ABS046 | 7 | 7 | 7 | 7 | 7 | 7 | 7 | 12 | 7 | 7 | 7 | 7 | 12 | 7 | 7 | 15 | 7 | 7 | 7 | 16 | 15 | 7 | 7 | 7 | 7 | 16 | | 10 | | 7 |
| ABS064 | 7 | 7 | 13 | 7 | 7 | 7 | 7 | 12 | 7 | 7 | 16 | 7 | 10 | 7 | 19 | 18 | 7 | 7 | 7 | 15 | 16 | 7 | 7 | 7 | 7 | 15 | | 7 | | 10 |
| ABS078 | 7 | 7 | 7 | 7 | 8 | 7 | 7 | 10 | 7 | 7 | 7 | 7 | 13 | 7 | 7 | 14 | 7 | 7 | 7 | 16 | 7 | 7 | 7 | 8 | 7 | 21 | | 11 | | 10 |
| ABS081 | 7 | 7 | 13 | 7 | 12 | 7 | 7 | 12 | 7 | 7 | 11 | 7 | 15 | 7 | 7 | 18 | 7 | 7 | 7 | 16 | 15 | 7 | 7 | 8 | 7 | 19 | | 12 | | 13 |
| ABS084 | 7 | 7 | 8 | 7 | 7 | 7 | 7 | 7 | 7 | 7 | 16 | 7 | 8 | 7 | 19 | 15 | 7 | 7 | 7 | 16 | 19 | 7 | 7 | 7 | 7 | 19 | | 7 | | 11 |
| ABS086 | 7 | 7 | 13 | 7 | 12 | 7 | 7 | 13 | 7 | 7 | 11 | 7 | 15 | 7 | 7 | 20 | 7 | 7 | 7 | 15 | 15 | 7 | 7 | 8 | 7 | 19 | | 18 | | 12 |
| ABS087 | 7 | 7 | 7 | 7 | 7 | 7 | 7 | 10 | 7 | 7 | 7 | 7 | 14 | 7 | 7 | 14 | 7 | 7 | 7 | 15 | 18 | 7 | 7 | 7 | 7 | 18 | | 13 | | 9 |
| ABS115 | 7 | 7 | 7 | 7 | 12 | 7 | 7 | 9 | 7 | 7 | 7 | 7 | 11 | 7 | 7 | 14 | 7 | 7 | 7 | 14 | 12 | 7 | 7 | 10 | 7 | 17 | | 18 | | 11 |
| ABS122 | 7 | 7 | 7 | 7 | 15 | 7 | 7 | 8 | 7 | 7 | 7 | 7 | 7 | 7 | 17 | 15 | 7 | 7 | 7 | 15 | 19 | 7 | 7 | 7 | 7 | 19 | | 21 | | 12 |
| ABS138 | 7 | 7 | 7 | 7 | 13 | 7 | 7 | 8 | 7 | 7 | 7 | 7 | 12 | 7 | 7 | 14 | 7 | 7 | 7 | 15 | 12 | 7 | 7 | 10 | 7 | 18 | | 16 | | 10 |
| ABS155 | 7 | 7 | 8 | 7 | 14 | 7 | 7 | 9 | 7 | 7 | 7 | 7 | 11 | 7 | 7 | 15 | 7 | 7 | 7 | 18 | 7 | 7 | 7 | 7 | 7 | 15 | | 15 | | 7 |
| ABS180 | 7 | 7 | 7 | 7 | 7 | 7 | 7 | 8 | 7 | 7 | 16 | 7 | 9 | 7 | 7 | 15 | 7 | 7 | 7 | 16 | 12 | 7 | 7 | 7 | 7 | 13 | | 10 | | 10 |
| ABS186 | 7 | 7 | 7 | 7 | 7 | 7 | 7 | 8 | 7 | 7 | 7 | 7 | 7 | 7 | 7 | 14 | 7 | 7 | 7 | 15 | 7 | 7 | 7 | 10 | 7 | 16 | | 14 | | 9 |
| ABS206 | 7 | 7 | 12 | 7 | 13 | 7 | 7 | 14 | 7 | 7 | 7 | 7 | 14 | 7 | 7 | 17 | 7 | 7 | 7 | 15 | 15 | 7 | 7 | 9 | 7 | 15 | | 17 | | 12 |
| ABS216 | 7 | 7 | 7 | 7 | 10 | 7 | 7 | 10 | 7 | 7 | 7 | 7 | 10 | 7 | 7 | 15 | 7 | 7 | 7 | 15 | 12 | 7 | 7 | 10 | 7 | 14 | | 19 | | 11 |
| ABS224 | 7 | 7 | 7 | 7 | 7 | 7 | 7 | 8 | 7 | 7 | 15 | 7 | 10 | 7 | 17 | 15 | 7 | 7 | 7 | 16 | 20 | 7 | 7 | 11 | 7 | 16 | | 11 | | 9 |
| ABS226 | 7 | 7 | 7 | 7 | 10 | 7 | 7 | 7 | 7 | 7 | 7 | 7 | 7 | 7 | 7 | 13 | 7 | 7 | 7 | 16 | 12 | 7 | 7 | 10 | 7 | 18 | | 18 | | 10 |
| ABS230 | 7 | 7 | 13 | 7 | 12 | 7 | 7 | 13 | 7 | 7 | 7 | 7 | 17 | 7 | 7 | 20 | 7 | 7 | 7 | 15 | 12 | 7 | 7 | 9 | 7 | 19 | | 17 | | 14 |
| ABS256 | 7 | 7 | 7 | 7 | 7 | 7 | 7 | 8 | 7 | 7 | 15 | 7 | 9 | 7 | 19 | 14 | 7 | 7 | 7 | 15 | 17 | 7 | 7 | 11 | 7 | 15 | | 10 | | 9 |
| ABS258 | 7 | 7 | 7 | 7 | 13 | 7 | 7 | 9 | 7 | 7 | 7 | 7 | 13 | 7 | 7 | 15 | 7 | 7 | 7 | 16 | 12 | 7 | 7 | 12 | 8 | 17 | | 15 | | 8 |
| ABS263 | 7 | 7 | 7 | 7 | 10 | 7 | 7 | 10 | 7 | 7 | 7 | 7 | 14 | 7 | 7 | 15 | 7 | 7 | 7 | 16 | 14 | 7 | 7 | 13 | 8 | 18 | | 16 | | 7 |
| ABS274 | 7 | 7 | 7 | 7 | 7 | 7 | 7 | 7 | 7 | 7 | 7 | 7 | 11 | 7 | 17 | 15 | 7 | 7 | 7 | 15 | 13 | 7 | 7 | 7 | 7 | 16 | | 7 | | 10 |
| ABS278 | 7 | 7 | 8 | 7 | 10 | 7 | 7 | 9 | 8 | 7 | 7 | 7 | 10 | 7 | 7 | 15 | 7 | 7 | 7 | 16 | 17 | 7 | 7 | 7 | 7 | 18 | | 10 | | 7 |
| ABS285 | 7 | 7 | 7 | 7 | 18 | 7 | 7 | 8 | 7 | 7 | 7 | 7 | 14 | 7 | 7 | 14 | 10 | 7 | 12 | 18 | 12 | 7 | 7 | 10 | 7 | 18 | | 7 | | 7 |
| ABS294 | 7 | 7 | 7 | 7 | 8 | 7 | 7 | 7 | 7 | 7 | 7 | 7 | 7 | 7 | 7 | 13 | 10 | 7 | 7 | 16 | 7 | 7 | 10 | 12 | 7 | 16 | | 12 | | 10 |
| ABS124 | 7 | 7 | 7 | 7 | 7 | 7 | 7 | 8 | 7 | 7 | 16 | 7 | 8 | 7 | 18 | 15 | 7 | 7 | 7 | 15 | 17 | 7 | 7 | 7 | 7 | 15 | | 12 | | 10 |
| ABS201 | 7 | 7 | 7 | 7 | 7 | 7 | 7 | 7 | 7 | 7 | 18 | 7 | 10 | 7 | 19 | 15 | 9 | 7 | 7 | 17 | 19 | 7 | 7 | 11 | 7 | 16 | | 12 | | 10 |
| ABS237 | 7 | 7 | 7 | 7 | 11 | 7 | 7 | 9 | 7 | 7 | 7 | 7 | 13 | 7 | 7 | 14 | 7 | 7 | 7 | 19 | 18 | 7 | 7 | 13 | 8 | 16 | | 15 | | 7 |

* Ap: Ampicillin, Sm: Streptomycin, Sp: Spectinomycin, Su: Sulfamethoxazole, Tc: Tetracycline, Tp: Trimethoprim, Km: Kanamycin, Nm: Neomycin, CTX: Cefotaxime, CAZ: Ceftazidime, Gm: Gentamicin, Cip: Ciprofloxacin, AK: Amikacin, Nx: Nalidixic Acid, Tm: Tobramycin, Ne: Netilmicin, Ipm: Imipenem, Mem: Meropenem, TIM: Timentin (Ticarcillin/clavulanic acid), Rif: Rifampicin, SAM: Ampicillin/sulbactam, FEP: Cefepime, DOR: Doripenem, TZP: Piperacillin/Tazobactam, CRO: Ceftriaxone, MIN: Minocycline, DOX: Doxycycline, LVX: Levofloxacin. Inhibition zone diameters highlighted white, light gray and dark gray indicate susceptibility, intermediate susceptibility, and resistance, respectively. White indicates susceptibility, dark grey reduced susceptibility and dark grey resistance.
